# Acute glycemic variability and short-term mortality of patients with subarachnoid hemorrhage: a meta-analysis

**DOI:** 10.3389/fneur.2026.1821856

**Published:** 2026-05-13

**Authors:** Lin Huang, Le Xie, Dahua Wu

**Affiliations:** 1 Graduate School, Hunan University of Chinese Medicine, Changsha, China; 2 Department of Neurology, Hunan Academy of Chinese Medicine Affiliated Hospital, Changsha, China

**Keywords:** glucose fluctuation, glycemic variability, meta-analysis, mortality, subarachnoid hemorrhage

## Abstract

**Background:**

Acute glycemic variability (GV) has been proposed as a potential prognostic marker in critically ill patients, but its association with mortality in subarachnoid hemorrhage (SAH) remains unclear. We conducted a meta-analysis to evaluate the relationship between acute GV and short-term mortality in patients with SAH.

**Methods:**

PubMed, Embase, Web of Science, Wanfang, and CNKI were searched from inception to identify longitudinal observational studies assessing acute GV during hospitalization and reporting short-term mortality (≤90 days) in adult patients with SAH. Odds ratios (ORs) with 95% confidence intervals (CIs) were pooled using random-effects models accounting for the influence of potential heterogeneity.

**Results:**

Seven cohort studies involving 10,119 patients were included, among whom 2,485 (24.6%) died within 90 days. Pooled results suggested that high acute GV was significantly associated with increased short-term mortality (OR 1.64, 95% CI 1.34–2.01; *p* < 0.001; *I*^2^ = 15%). Subgroup analyses showed a stronger association was observed in studies with glucose monitoring > 3 days compared with ≤3 days (OR 2.62 vs. 1.48; *p* for subgroup difference = 0.03). Further subgroup analyses suggested that the association was consistent across subgroups stratified by study design, geographic region, mean age, sex distribution, diabetes proportion, follow-up duration, and study quality (all *p* for subgroup differences > 0.05).

**Conclusion:**

Higher acute GV was associated with increased short-term mortality in patients with SAH. Prolonged glucose monitoring may enhance prognostic value. These findings suggest that acute glucose fluctuations may serve as a risk factor for short-term mortality in patients with SAH.

**Systematic review registration:**

https://www.crd.york.ac.uk/prospero/search, identifier CRD420261330574.

## Introduction

Subarachnoid hemorrhage (SAH) is a severe and life-threatening subtype of stroke characterized by bleeding into the subarachnoid space, most commonly resulting from rupture of an intracranial aneurysm (1, 2). Although SAH accounts for a relatively small proportion of all strokes, it carries disproportionately high mortality and long-term disability, frequently affecting individuals at a younger age compared with other stroke subtypes (3, 4). Despite advances in early aneurysm repair and neurocritical care, short-term case-fatality rates remain substantial, particularly within the first weeks after onset (5, 6). Survivors often experience persistent neurological deficits, cognitive impairment, and reduced quality of life (7, 8). Established predictors of mortality after SAH include advanced age, poor neurological status at admission (e.g., high Hunt and Hess or WFNS grades), extensive hemorrhage burden, delayed cerebral ischemia, and systemic complications (9, 10). In recent years, metabolic disturbances—especially dysglycemia—have also emerged as potentially important contributors to adverse outcomes in this population (11).

Acute glycemic variability (GV) refers to short-term fluctuations in blood glucose levels occurring over hours to days and is typically quantified using indices such as standard deviation (SD), coefficient of variation (CV), mean amplitude of glycemic excursions (MAGE), or other dynamic metrics derived from serial glucose measurements (12, 13). Unlike isolated glucose values, GV captures instability in glucose homeostasis and may better reflect metabolic stress during acute illness (14). Fluctuating glucose levels have been associated with increased oxidative stress, inflammatory activation, endothelial dysfunction, and impaired cerebral autoregulation, mechanisms that may aggravate secondary brain injury and contribute to poor prognosis after SAH (15, 16). Several observational studies have investigated the association between acute GV and mortality in patients with SAH (17–23). However, variations in study design, sample size, and GV assessment methods limit the interpretability of individual findings. Therefore, we conducted a systematic review and meta-analysis of longitudinal studies to evaluate the association between acute glycemic variability and short-term mortality in patients with SAH and to explore potential modifiers of this relationship.

## Methods

The meta-analysis was carried out in accordance with established methodological guidance, following the principles outlined in the PRISMA 2020 statement (24) and the Cochrane Handbook for Systematic Reviews and Meta-Analyses (25), encompassing protocol planning, study selection, data extraction, statistical analysis, and reporting. The study protocol was registered prospectively in the PROSPERO database (registration number: CRD420261330574).

### Database search

We carried out a comprehensive literature search across PubMed, Embase, Web of Science, Wanfang, and China National Knowledge Infrastructure (CNKI) to identify eligible studies for inclusion. The search strategy was constructed using the combined terms of (1) “glycemic variability” OR “glyceamic variability” OR “glucose variability” OR “glucose fluctuation” OR “standard deviation of blood glucose” OR “coefficient of variation of blood glucose” OR “glycemic lability index” OR “GLI” OR “mean amplitude of glycemic excursion” OR “MAGE” OR “largest amplitude of glycemic excursion” OR “LAGE”; and (2) “subarachnoid hemorrhage” OR “subarachnoid hemorrhage” OR “SAH.” Only full-text, peer-reviewed articles published in English or Chinese and conducted in human populations were considered eligible. We also manually examined the reference lists of relevant reviews and original studies to capture additional potentially eligible reports. Each database was searched from inception through 22 January 2026. The complete search strategies for all databases are provided in Supplementary File 1.

### Study inclusion and exclusion criteria

Studies were considered eligible if they met the following inclusion criteria: (1) enrolled adult patients (≥18 years) diagnosed with SAH, confirmed by neuroimaging or cerebrospinal fluid examination; (2) assessed acute GV during the early phase of hospitalization (e.g., ICU stay or initial admission) using established or commonly used indices, such as SD, CV, MAGE), glycemic lability index (GLI, or other recognized metrics; (3) compared patients with higher versus lower GV according to study-specific definitions; (4) reported short-term mortality outcomes (≤90 days); and (5) were observational longitudinal studies, including prospective or retrospective cohort studies, nested case–control studies, or *post hoc* analyses of clinical studies.

Studies were excluded if they met any of the following criteria: (1) cross-sectional studies, case reports, case series, reviews, meta-analyses, editorials, letters, or studies conducted in animals or *in vitro*; (2) did not specifically include patients with SAH or did not provide extractable data for SAH patients; (3) did not assess GV during the acute phase or evaluated only mean glucose levels without variability metrics; (4) did not report short-term mortality outcomes or lacked longitudinal follow-up; (5) did not provide sufficient data to estimate effect sizes; or (6) involved duplicate or overlapping populations (in which case the study with the largest sample size or most comprehensive data was included).

### Study quality evaluation and data extraction

Two reviewers independently performed the literature search, screened eligible studies, assessed study quality, and extracted relevant data. Any disagreements were resolved through discussion, and when necessary, by consulting the corresponding author. Study quality was appraised using the Newcastle–Ottawa Scale (NOS) (26). The NOS examines methodological rigor across selection, comparability, and outcome ascertainment domains. Total scores vary from 1 to 9, and studies achieving ≥ 7 points were considered to be of high quality. Extracted data included study characteristics (first author, publication year, country, and study design), participant characteristics (diagnosis, sample size, age, sex, and diabetic status), exposure assessment (timing and parameters used to evaluate GV, and cutoffs for defining high GV), follow-up duration, number of patients who died during follow-up, and covariates included in the adjusted analyses examining the association between GV and short-term mortality of patients with SAH.

### Statistical analyses

Although different indices were used to quantify GV (e.g., SD, CV, MAGE, and other derived metrics), these measures are generally considered to reflect a common underlying construct of short-term glucose fluctuation (13, 27). Therefore, consistent with prior literature, we treated different GV indices as comparable indicators of glycemic instability and pooled the corresponding effect estimates. The association between GV and short-term mortality of patients with SAH was evaluated by combining odds ratios (ORs) and their corresponding 95% confidence intervals (CIs), comparing individuals with high vs. low GV. When necessary, effect estimates and standard errors were derived from reported 95% CIs or *p* values. All estimates were log-transformed before pooling to enhance normal distribution assumptions and stabilize variances (25). To evaluate variability across studies, we applied the Cochrane *Q* test and calculated the *I*^2^ statistic (28). *I*^2^ values below 25% were classified as low heterogeneity, 25–75% as moderate, and above 75% as high heterogeneity. Pooled effect estimates were calculated using a random-effects model to accommodate variability across studies (25). We performed leave-one-out sensitivity analyses, sequentially excluding individual studies to assess the robustness of the findings (29). To identify possible sources of between-study variability, we performed predefined subgroup analyses stratified by study design (prospective or retrospective), country (Asian vs. non-Asian), mean age, sex distribution, proportion of patients with diabetes, duration of blood glucose monitoring for the evaluation of GV, follow-up durations, and study quality scores. Continuous variables were dichotomized using their median values as cutoffs to balance the number of studies in each subgroup. Subgroup analysis according to GV metric type was not performed because most indices were reported in only one study, precluding meaningful statistical comparison. To assess potential publication bias, we visually inspected funnel plots for asymmetry. Egger’s regression test was also performed as an exploratory analysis (30). However, given the small number of included studies (<10), its statistical power is limited and the results should be interpreted with caution (30). A two-tailed *p* value < 0.05 was considered statistically significant. All statistical analyses were performed using RevMan (version 5.3; Cochrane Collaboration, Oxford, United Kingdom) and Stata (version 17.0; StataCorp, College Station, TX, United States).

## Results

### Database search results

**Figure 1:**
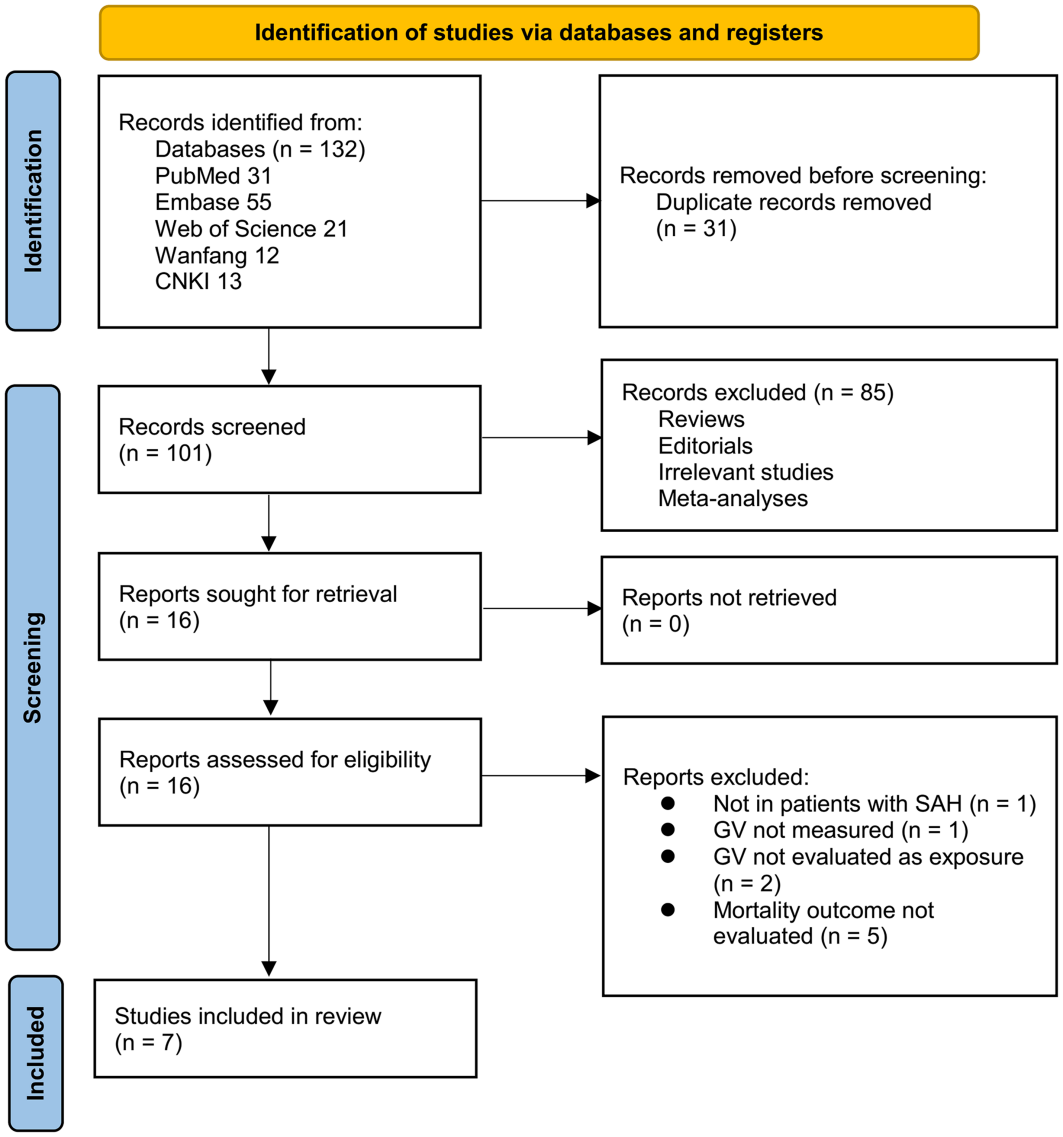
Flow diagram of the study selection process.

The study selection procedure is illustrated in Figure 1. A total of 132 records were retrieved from the five databases, and 31 duplicates were removed. Following screening of titles and abstracts, 85 records were excluded for failing to meet the predefined inclusion criteria. Sixteen articles underwent full-text evaluation by two independent reviewers, after which nine studies were excluded for the reasons detailed in Figure 1. Ultimately, seven studies met the eligibility criteria and were included in the quantitative meta-analysis (17–23).

### Summary of study characteristics

Table 1 summarizes the characteristics of the included cohort studies. A total of seven cohort studies were incorporated, including two prospective cohorts (18, 21) and five retrospective cohorts (17, 19, 20, 22, 23). These studies were published between 2014 and 2025 and were conducted across the United States, China, and Australia/New Zealand. The included populations comprised patients with spontaneous or aneurysmal SAH, primarily managed in intensive care units (ICU) (18–20, 23). Sample sizes varied markedly, ranging from 28 to 6,098 patients, with a total of 10,119 participants with SAH across studies. Mean age ranged from 47.3 to 62.7 years, and the proportion of men varied between 28.7 and 64.5%. GV was assessed during the acute hospitalization phase, most commonly immediately upon hospital or ICU admission. Various GV indices were used, including standard deviation of blood glucose (SDBG) (17), MAGE (18), coefficient of variation of blood glucose (CVBG) (21, 23), mean consecutive absolute change percentage (MCACP%) (20), maximal blood glucose difference (Q5: Q1) (19), and predefined fasting blood glucose fluctuation patterns (22). The duration of blood glucose monitoring ranged from 1 to 14 days. Follow-up periods ranged from in-hospital assessment to 90 days, and the number of deaths reported in individual studies ranged from 5 to 1,508. Accordingly, a total of 2,485 (24.6%) patients died within 3 months of SAH onset. Six studies (18–23) adjusted for important confounders such as age, sex, and disease severity scores (e.g., Acute Physiology and Chronic Health Evaluation II Scores, and Hunt and Hess grades), comorbidities (e.g., diabetes, hypertension, coronary artery disease), and treatment-related variables to a varying degree. One study (17) only reported crude OR and 95% CI for the association between high GV and risk of short-term mortality of patients with SAH.

**Table 1:** Characteristics of the included cohort studies.

Study	Design	Location	Diagnosis	No. of patient	Mean age (years)	Men (%)	DM (%)	Timing for evaluation of GV	GV indices and cutoffs	Duration of BG measuring for evaluating GV (days)	Follow-up duration (days)	No. of patients died	Variables adjusted
Kurtz et al. (2014) (17)	RC	USA	Spontaneous SAH (poor-grade, GCS ≤ 8)	28	54.0	32.0	11	During the neuromonitoring period, starting a median of 2 days after admission	SDBG, median (1.4 mmol/L)	6	In-hospital	7	None
Zhang et al. (2018) (18)	PC	China	aSAH	65	57.3	55.4	0	Immediately upon ICU admission	MAGE, ROC curve analysis derived (3.69 mmol/L)	3	28	27	Age, sex, 24 h APACHE II score, and history of hypertension
Pappacena et al. (2019) (19)	RC	Australia and New Zealand	SAH	6,098	47.3	64.5	NR	Immediately upon ICU admission	Maximal BG difference in the first day, Q5: Q1 (4.6 mmol/L)	1	In-hospital	1,508	Age, year of admission, ventilation status, patient severity, and study site
Sadan et al. (2020) (20)	RC	USA	aSAH	2,451	53.0	28.7	9.4	Immediately upon ICU admission	MCACP%, median (14.5%)	5	90	694	Age, sex, HH grade, smoking, hypertension, CAD, hyperlipidemia, DM, and treatments
Xu et al. (2020) (21)	PC	China	SAH	80	62.7	46.3	0	Immediately upon admission	CVBG, previous study determined (50%)	3	28	5	Age, sex, BMI, APACHE II score, and HOMA-IR
Wu et al. (2022) (22)	RC	China	aSAH	341	55.6	43.4	15.2	Immediately upon admission	FBG daily difference: High GV: The unstable group (Day 1 < 7 mmol/L, Days 2–14 at least one ≥10 mmol/L), otherwise low GV (Day 1 < 7 mmol/L, Days 2–14 all <10 mmol/L)	14	28	17	Age, sex, HH grade, admission SBP, BUN, d-dimer, HbA1c, and IVH
Hou et al. (2025) (23)	RC	USA	Non-traumatic SAH	1,056	61.0	44.4	16.4	Immediately upon ICU admission	CVBG, T3: T1 (20.4%)	3	90	227	Age, sex, ethnicity, HR, MAP, SaO2, CRRT, MV, DM status, and comorbidities

RC, retrospective cohort; PC, prospective cohort; SAH, subarachnoid hemorrhage; aSAH, aneurysmal subarachnoid hemorrhage; GCS, Glasgow Coma Scale; ICU, intensive care unit; GV, glycemic variability; BG, blood glucose; SDBG, standard deviation of blood glucose; MAGE, mean amplitude of glycemic excursions; ROC, receiver operating characteristic; Q5: Q1, fifth versus first quintile; MCACP%, mean consecutive absolute change percentage; CVBG, coefficient of variation of blood glucose; FBG, fasting blood glucose; DM, diabetes mellitus; APACHE II, Acute Physiology and Chronic Health Evaluation II; HH grade, Hunt and Hess grade; CAD, coronary artery disease; BMI, body mass index; HOMA-IR, homeostasis model assessment of insulin resistance; SBP, systolic blood pressure; BUN, blood urea nitrogen; HbA1c, glycated hemoglobin; IVH, intraventricular hemorrhage; HR, heart rate; MAP, mean arterial pressure; SaO2, arterial oxygen saturation; CRRT, continuous renal replacement therapy; MV, mechanical ventilation; NR, not reported.

### Study quality evaluation

Methodological quality was assessed using the NOS, and the detailed results are presented in Table 2. The included studies achieved NOS scores ranging from 7 to 9, indicating generally high methodological quality. Five studies (18, 20–23) achieved the maximum score of 9, reflecting strong cohort selection, adequate control of confounding (including age and additional variables), reliable exposure ascertainment, appropriate outcome assessment, and sufficient follow-up. One study (19) scored 8, primarily due to limited representativeness of the exposed cohort. Another study (17) received a score of 7, mainly because of limited adjustment for confounders. Overall, all included studies were considered to be of acceptable to high quality, supporting the robustness of the pooled estimates evaluating the prognostic value of acute GV in patients with SAH.

**Table 2:** Study quality evaluation via the Newcastle–Ottawa Scale.

Study	Representativeness of the exposed cohort	Selection of the non-exposed cohort	Ascertainment of exposure	Outcome not present at baseline	Control for age	Control for other confounding factors	Assessment of outcome	Enough long follow-up duration	Adequacy of follow-up of cohort	Total
Kurtz et al. (2014) (17)	1	1	1	1	0	0	1	1	1	7
Zhang et al. (2018) (18)	1	1	1	1	1	1	1	1	1	9
Pappacena et al. (2019) (19)	0	1	1	1	1	1	1	1	1	8
Sadan et al. (2020) (20)	1	1	1	1	1	1	1	1	1	9
Xu et al. (2020) (21)	1	1	1	1	1	1	1	1	1	9
Wu et al. (2022) (22)	1	1	1	1	1	1	1	1	1	9
Hou et al. (2025) (23)	1	1	1	1	1	1	1	1	1	9

### Meta-analysis results

**Figure 2:**
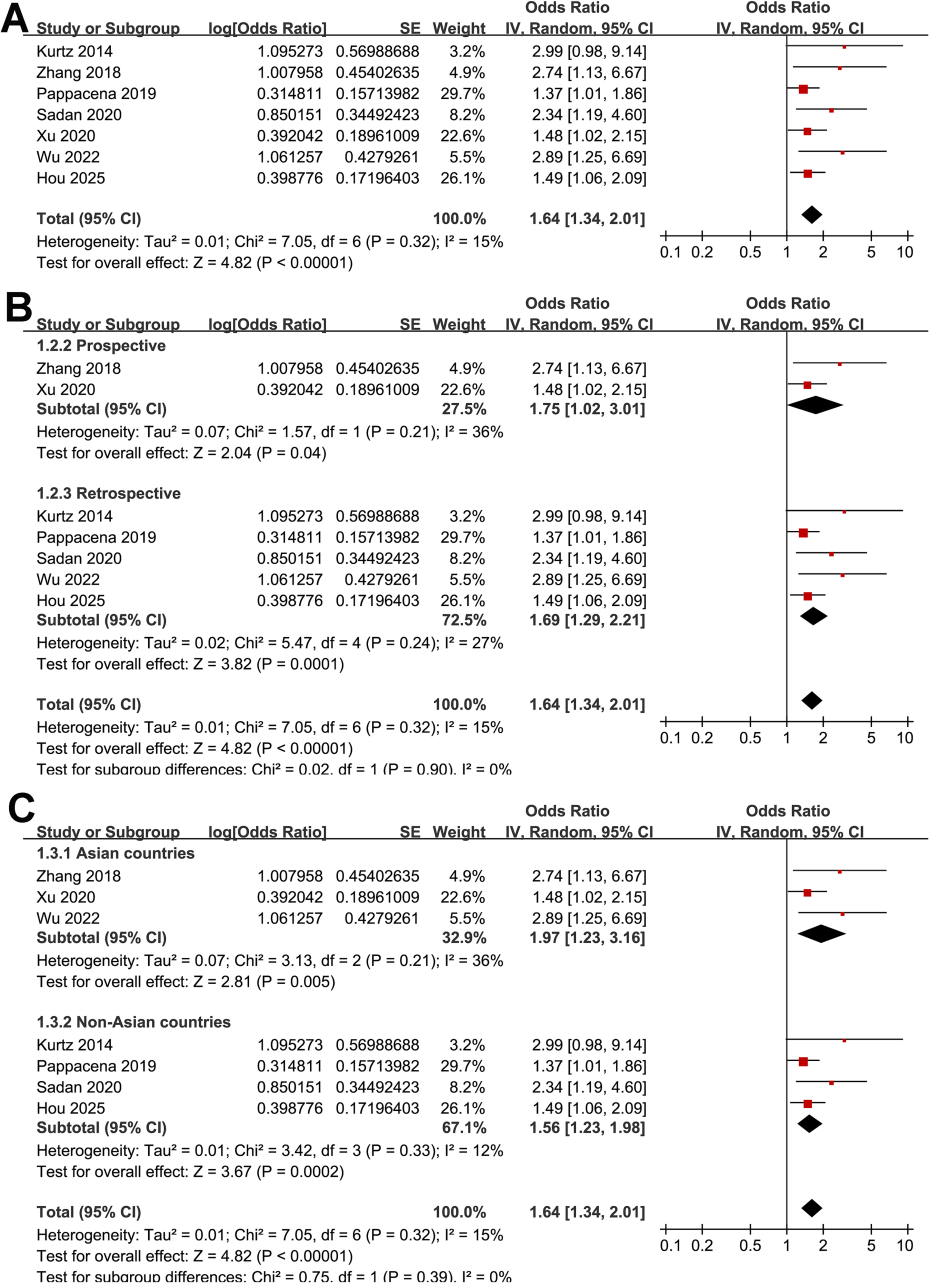
Forest plots for the meta-analysis of the association between acute GV and short-term mortality of patients with SAH; **(A)**, overall meta-analysis; **(B)** subgroup analysis by study design; and **(C)** subgroup analysis by study country.

**Figure 3:**
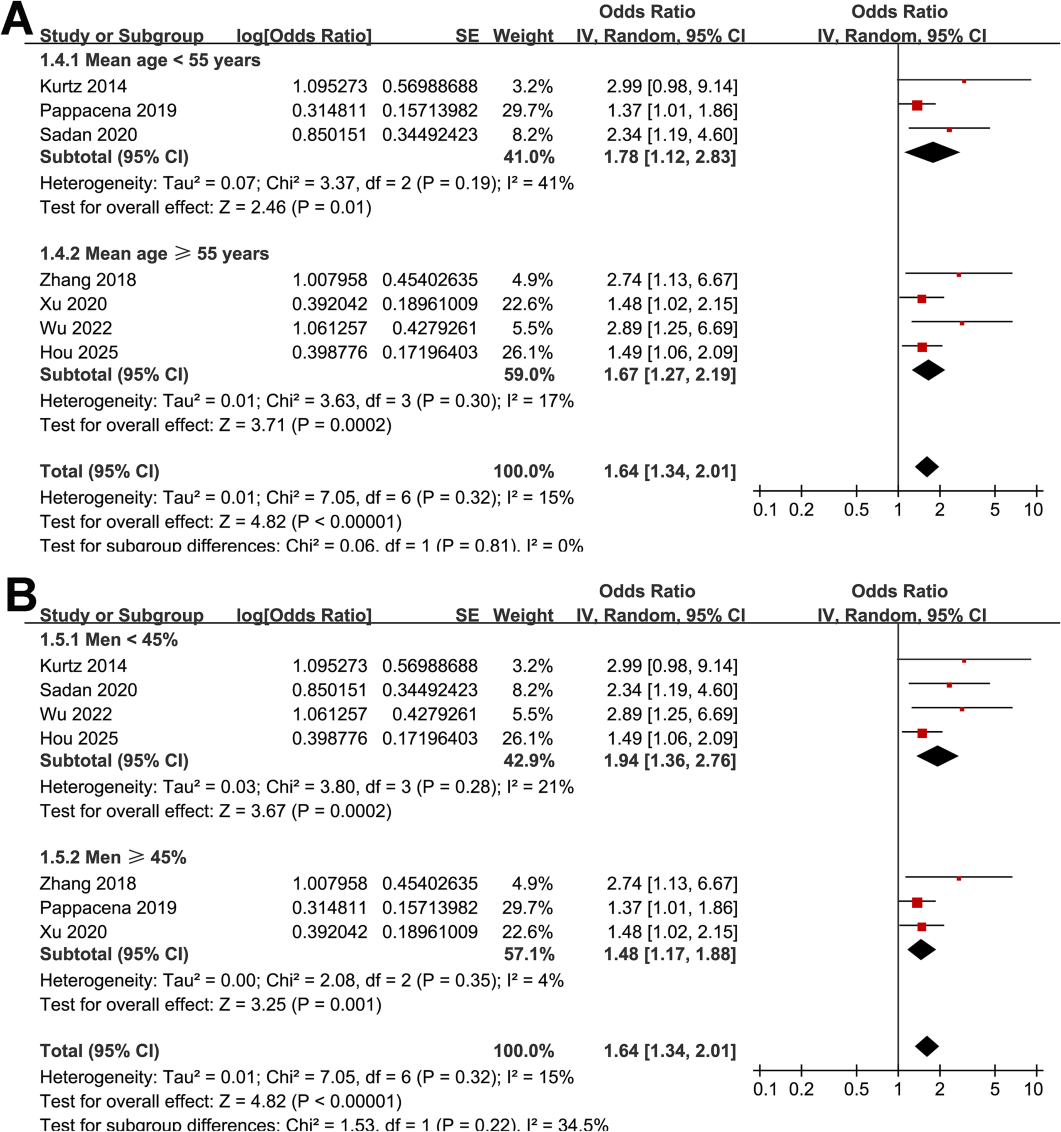
Forest plots for subgroup analyses of the association between acute GV and short-term mortality of patients with SAH; **(A)** stratified by mean ages of the patients; **(B)** stratified by proportions of men.

**Figure 4:**
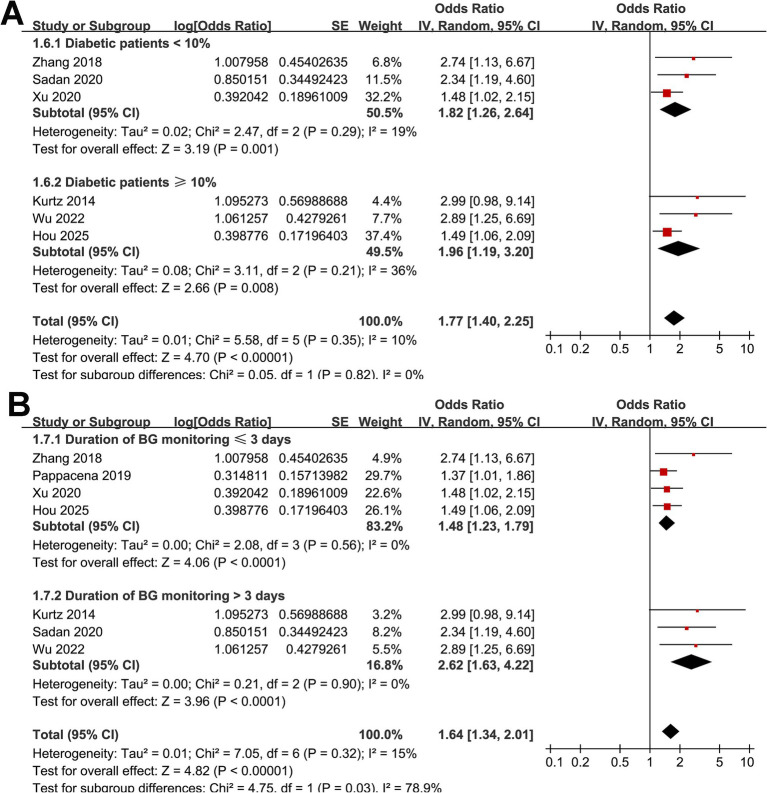
Forest plots for subgroup analyses of the association between acute GV and short-term mortality of patients with SAH; **(A)** stratified by proportion of diabetic patients; **(B)** stratified by duration of glycemic monitoring for evaluating GV.

**Figure 5:**
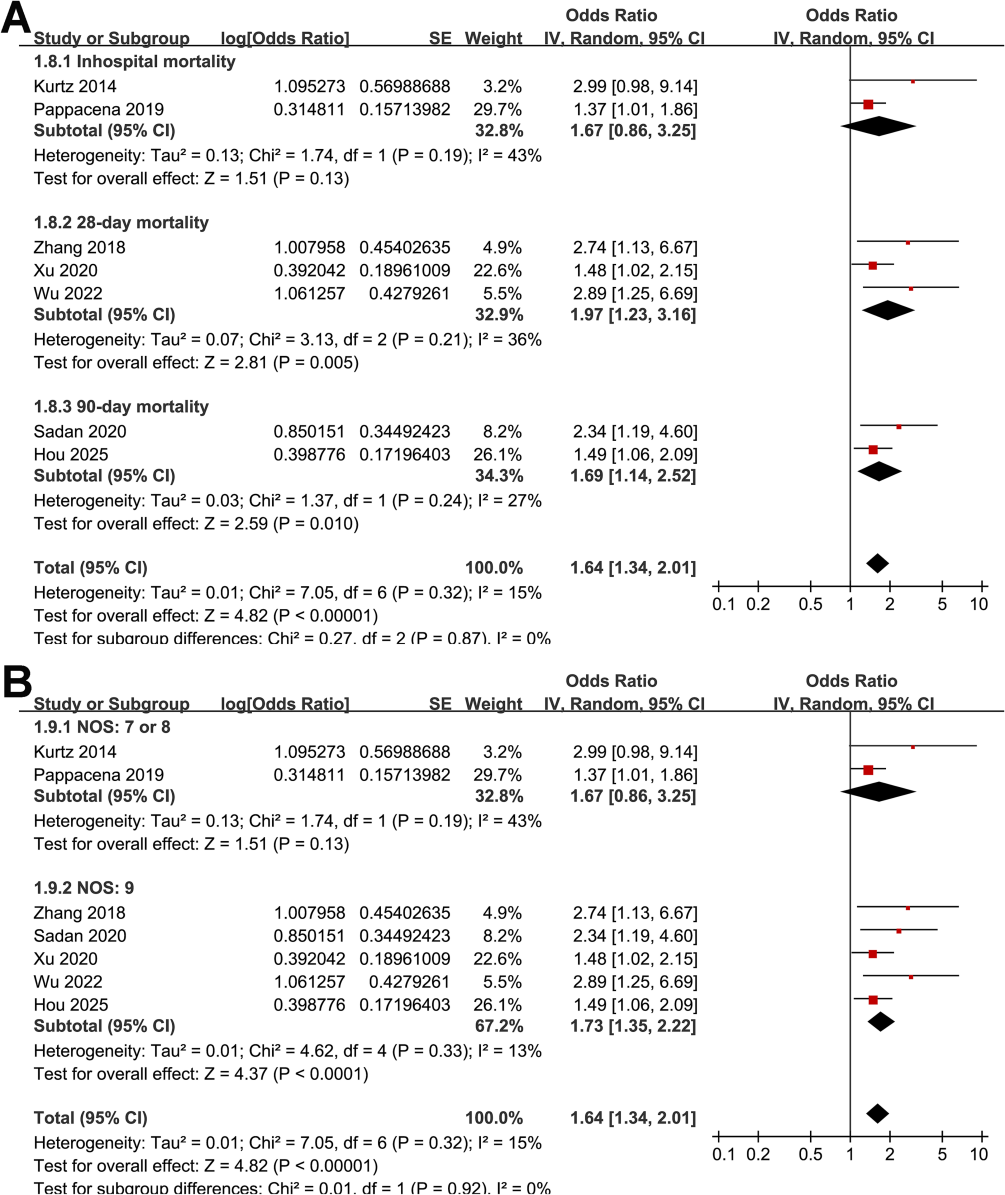
Forest plots for subgroup analyses of the association between acute GV and short-term mortality of patients with SAH; **(A)** stratified by follow-up duration; **(B)** stratified by study quality scores of NOS.

Across seven cohort studies (17–23), the pooled results of the meta-analysis demonstrated that high acute GV in adults with SAH was associated with a significantly increased risk of short-term mortality within 90 days after disease onset (OR: 1.64, 95% CI: 1.34–2.01; *p* < 0.001; Figure 2A) with mild heterogeneity (*p* for Cochrane *Q* test = 0.32, *I*^2^ = 15%). Removing studies one at a time did not substantially alter the overall results, with pooled ORs ranging from 1.56 to 1.77 (all *p* < 0.05). Specifically, excluding the only study with univariate analysis showed consistent results (OR: 1.61, 95% CI: 1.31–1.96; *p* < 0.001; *I*^2^ = 14%). Subgroup analyses yielded generally consistent findings across predefined study characteristics. The association between high acute GV and short-term mortality of patients with SAH did not differ significantly between prospective and retrospective cohorts (OR: 1.75 vs. 1.69; *p* for subgroup difference = 0.90; Figure 2B), between studies from Asian and non-Asian countries (OR: 1.97 vs. 1.56; *p* for subgroup difference = 0.39; Figure 2C), between studies with the mean ages of the patients <55 and ≥55 years (OR: 1.78 vs. 1.67; *p* for subgroup difference = 0.81; Figure 3A), between patients with the proportions of men < 45% and ≥ 45% (OR: 1.94 vs. 1.48; *p* for subgroup difference = 0.22; Figure 3B), and between studies with the proportions of patients with diabetes < 10% and ≥10% (OR: 1.82 vs. 1.96; *p* for subgroup difference = 0.82; Figure 4A). Interestingly, a stronger association between high acute GV and mortality was observed in studies with the duration of glucose monitoring for evaluating GV > 3 days as compared to those ≤3 days (OR: 2.62 vs. 1.48, *p* for subgroup difference = 0.03; Figure 4B), which may completely explain the source of heterogeneity (*I*^2^ in both subgroups = 0%). Further subgroup analysis showed similar results between studies reporting in hospital, 28-day, and 90-day mortality (OR: 1.67, 1.97 vs. 1.69, *p* for subgroup difference = 0.87; Figure 5A), and in studies with the NOS score of 7 or 8, and 9 (OR: 1.67 vs. 1.73; *p* for subgroup difference = 0.92; Figure 5B).

### Publication bias

**Figure 6:**
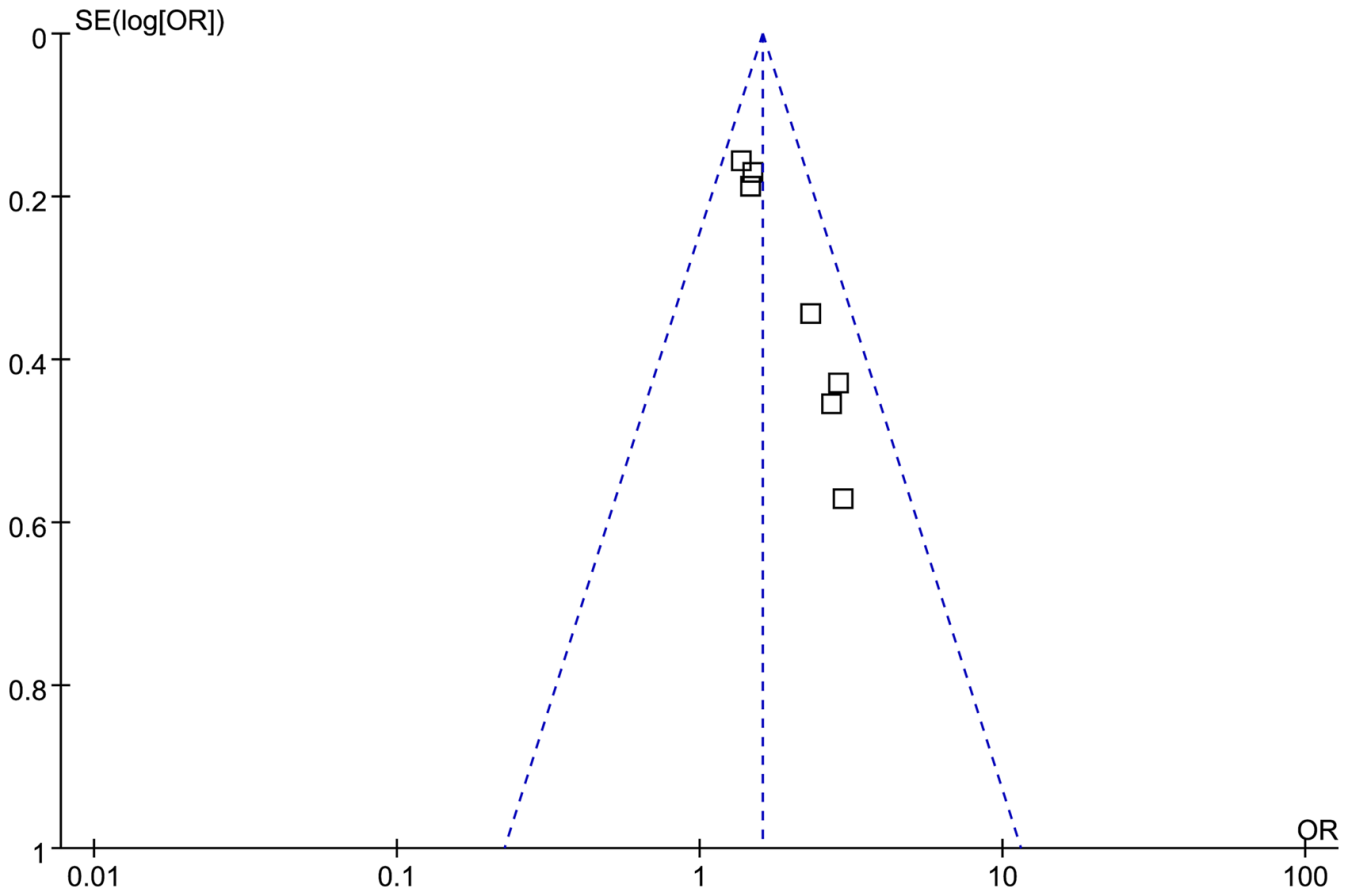
Funnel plots evaluating potential publication bias of the association between acute GV and short-term mortality of patients with SAH.

As shown in Figure 6, the funnel plots for the association between high acute GV and short-term mortality of patients with SAH appeared largely symmetrical. However, given the small number of included datasets (*n* = 7), formal statistical tests for publication bias are underpowered and should be interpreted cautiously. Egger’s test did not indicate statistically significant asymmetry (*p* = 0.39), but this result does not exclude the possibility of publication bias.

## Discussion

This meta-analysis demonstrated that increased acute GV is associated with higher short-term mortality in patients with SAH, reinforcing the clinical relevance of dynamic glucose instability during the acute phase of the disease. Beyond isolated hyperglycemia, our findings emphasize that fluctuations in glucose levels may carry independent prognostic significance. The robustness of the association across multiple subgroup and sensitivity analyses suggests that acute GV represents a consistent marker of adverse prognosis in SAH.

Several mechanistic pathways may explain the relationship between acute GV and mortality after SAH. Experimental and clinical evidence indicates that glucose fluctuations induce greater oxidative stress and endothelial dysfunction than sustained hyperglycemia alone (31, 32), thereby amplifying inflammatory cascades and microvascular injury. In severe spontaneous SAH, high glucose variability has been independently associated with cerebral infarction, presumably related to vasospasm, even after multivariable adjustment (33). In that study, patients with high variability had markedly higher rates of cerebral infarction (64% vs. 20%), and glucose variability remained an independent predictor (OR 11.44), supporting the hypothesis that unstable glucose dynamics may contribute to secondary ischemic injury (33). Similarly, dysregulated glucose indices have been linked to delayed cerebral ischemia (DCI), a major determinant of poor outcome (34). In a retrospective cohort of aneurysmal SAH, elevated time-weighted average glucose and dysglycemic rates were observed before and at the onset of DCI (34), suggesting that early metabolic instability may precede and potentially facilitate ischemic complications. From a clinical perspective, acute GV may also reflect systemic stress responses, catecholamine surges, autonomic dysfunction, and treatment-related glucose swings, all of which may aggravate early brain injury and impair cerebral autoregulation (35).

Emerging data using continuous glucose monitoring (CGM) further support the prognostic importance of dynamic glycemic metrics. In a prospective study evaluating SAH patients during the early brain injury phase, CGM-derived indices of glucose burden and variability were independently associated with poor 3-month functional outcomes (36). Notably, several variability-related parameters correlated with elevated neurofilament light chain levels, a biomarker of neuroaxonal injury (37), whereas conventional intermittent measurements showed weaker associations (36). These findings suggest that unstable glucose dynamics may be mechanistically linked to neuronal damage and long-term neurological impairment. Taken together, prior evidence indicates that glucose variability may contribute to secondary brain injury through oxidative stress, microvascular dysfunction, energy crisis, and promotion of ischemic complications, providing biological plausibility for the mortality association observed in our pooled analysis.

In subgroup analyses, a stronger association was observed in studies with glucose monitoring durations exceeding 3 days. This finding may reflect cumulative exposure to glycemic instability during the critical window of early brain injury and evolving complications such as vasospasm and DCI. Longer monitoring likely captures sustained metabolic dysregulation more accurately than short observation periods. The consistency of results across study design, geographic region, age, sex distribution, diabetes proportion, follow-up duration, and study quality further supports the generalizability of the association. Importantly, the lack of significant subgroup interactions suggests that the adverse impact of acute GV is not confined to specific patient subsets.

This meta-analysis has several strengths. We performed a comprehensive and up-to-date search across major international and Chinese databases, minimizing selection bias. All included studies were cohort designs with longitudinal follow-up, ensuring temporal sequencing between exposure and outcome. Most studies reported multivariable-adjusted estimates, accounting for important confounders such as age, disease severity scores, and comorbidities. Heterogeneity was low, and findings were robust across sensitivity analyses, enhancing confidence in the pooled results. Nevertheless, limitations should be acknowledged. First, most included studies were retrospective, which may introduce selection bias and restrict full adjustment for confounding (38). Second, heterogeneity existed in the parameters used to quantify GV and in the cutoff definitions for high versus low variability. Although commonly used indices such as SD, CV, and MAGE capture a shared construct of glucose fluctuation, differences in their mathematical properties may introduce measurement variability. Due to the limited number of studies using each specific metric, subgroup analysis by GV index was not feasible. Therefore, the potential influence of GV measurement methods on the observed association cannot be fully excluded. However, no universally accepted gold standard for GV assessment currently exists, and different indices reflect complementary aspects of glucose fluctuation (27). Differences in patient demographics, baseline neurological severity, treatment strategies, and comorbidities may also contribute to residual heterogeneity, which could not be fully explored due to the lack of individual participant data. Additionally, unmeasured factors, including insulin protocols, nutritional strategies, or other metabolic disturbances, may influence outcomes. As an observational synthesis, this study demonstrates association rather than definitive causation. Finally, the small number of included studies limited the ability to reliably assess publication bias.

From a clinical standpoint, our findings underscore the importance of recognizing and potentially stabilizing glucose fluctuations in the acute management of SAH. While current guidelines emphasize avoidance of extreme hyper- or hypoglycemia, greater attention to variability itself may enhance prognostic assessment and therapeutic optimization. Continuous or more frequent glucose monitoring may allow earlier identification of high-risk patients and provide a basis for targeted metabolic interventions. Future prospective studies and interventional trials are warranted to determine whether strategies aimed at reducing glycemic variability can improve survival and neurological outcomes.

## Conclusion

In conclusion, higher acute GV is associated with increased short-term mortality in patients with SAH. Accumulating mechanistic and clinical evidence suggests that unstable glucose dynamics may contribute to secondary ischemic injury and neuroaxonal damage. These findings highlight acute glucose fluctuation as a clinically meaningful prognostic factor in the neurocritical care of SAH.

## Data Availability

The original contributions presented in the study are included in the article/Supplementary material, further inquiries can be directed to the corresponding authors.
