# Supplementary material for: Acute glycemic variability and short-term mortality of patients with subarachnoid hemorrhage: a meta-analysis
Source: Front Neurol. 2026 May 13;17:1821856. doi: 10.3389/fneur.2026.1821856 (PMC13212109; doi:10.3389/fneur.2026.1821856)
Supplement: Supplementary file 1 [file Table_1.docx]

**Detailed search strategy for each database**

**PubMed**

#1 "Subarachnoid Hemorrhage"[Mesh] OR "subarachnoid hemorrhage"[tiab] OR "subarachnoid haemorrhage"[tiab] OR SAH[tiab]

#2 "Glycemic Variability"[tiab] OR "glyceamic variability"[tiab] OR "glucose variability"[tiab] OR "glucose fluctuation"[tiab] OR "standard deviation of blood glucose"[tiab] OR "coefficient of variation of blood glucose"[tiab] OR "glycemic lability index"[tiab] OR GLI[tiab] OR "mean amplitude of glycemic excursion"[tiab] OR MAGE[tiab] OR "largest amplitude of glycemic excursion"[tiab] OR LAGE[tiab]

#3 #1 AND #2

**Embase**

1. 'subarachnoid hemorrhage'/exp OR 'subarachnoid hemorrhage':ti,ab OR 'subarachnoid haemorrhage':ti,ab OR SAH:ti,ab

2. 'glycemic variability':ti,ab OR 'glyceamic variability':ti,ab OR 'glucose variability':ti,ab OR 'glucose fluctuation':ti,ab OR 'standard deviation of blood glucose':ti,ab OR 'coefficient of variation of blood glucose':ti,ab OR 'glycemic lability index':ti,ab OR GLI:ti,ab OR 'mean amplitude of glycemic excursion':ti,ab OR MAGE:ti,ab OR 'largest amplitude of glycemic excursion':ti,ab OR LAGE:ti,ab

3. 1 AND 2

**Web of Science**

TS=("subarachnoid hemorrhage" OR "subarachnoid haemorrhage" OR SAH) AND TS=("glycemic variability" OR "glyceamic variability" OR "glucose variability" OR "glucose fluctuation" OR "standard deviation of blood glucose" OR "coefficient of variation of blood glucose" OR "glycemic lability index" OR GLI OR "mean amplitude of glycemic excursion" OR MAGE OR "largest amplitude of glycemic excursion" OR LAGE)

**Wanfang**

主题: (“蛛网膜下腔出血” OR “SAH”) AND 主题: (“血糖变异” OR “葡萄糖变异” OR “葡萄糖波动” OR “血糖标准差” OR “血糖变异系数” OR “血糖波动指数” OR “GLI” OR “平均血糖波动幅度” OR “MAGE” OR “最大血糖波动幅度” OR “LAGE”)

**CNKI (China National Knowledge Infrastructure)**

主题: (“蛛网膜下腔出血” OR “SAH”) AND 主题: (“血糖变异” OR “葡萄糖变异” OR “葡萄糖波动” OR “血糖标准差” OR “血糖变异系数” OR “血糖波动指数” OR “GLI” OR “平均血糖波动幅度” OR “MAGE” OR “最大血糖波动幅度” OR “LAGE”)
